# A Time-Aware Routing Map for Indoor Evacuation †

**DOI:** 10.3390/s16010112

**Published:** 2016-01-18

**Authors:** Haifeng Zhao, Stephan Winter

**Affiliations:** Department of Infrastructure Engineering, The University of Melbourne, Parkville, Victoria 3010, Australia; winter@unimelb.edu.au

**Keywords:** indoor mapping, indoor evacuation, dynamic environments, decision making

## Abstract

Knowledge of dynamic environments expires over time. Thus, using static maps of the environment for decision making is problematic, especially in emergency situations, such as evacuations. This paper suggests a fading memory model for mapping dynamic environments: a mechanism to put less trust on older knowledge in decision making. The model has been assessed by simulating indoor evacuations, adopting and comparing various strategies in decision making. Results suggest that fading memory generally improves this decision making.

## 1. Introduction

The decision making of agents travelling in search of target destinations in a dynamic environment is a common research topic. The agents may be evacuees trying to find their way out of a building in the event of an emergency, or service robots moving towards a destination in an indoor environment with roaming kids and movable furniture, or robots collaboratively exploring unfamiliar terrain, or vehicles travelling on road networks with crossing pedestrians. The challenge for decision making is that such environments change all of the time. While learned and accumulated spatial knowledge may be trustworthy for decision making in (sufficiently) static environments, in dynamic environments, the past experience of the environment may form out-of-date knowledge. How to fulfil the wayfinding tasks of agents with potentially outdated knowledge is the research question addressed in this paper.

To date, the predominant evacuation strategies still rely on static signs, e.g., stationary exit signs [1] or you-are-here maps, which are of low efficiency [2] and are often perceived as confusing and unclear, especially when people are stressed or panicked [3]. Reliable shortest route planning in a dynamic environment is possible with a centralized, real-time sensing system that creates situation-aware pictures for each agent. However, such a centralized service will in most cases not exist due to the lack (or damage) of the required sensing and communication infrastructure. In this situation, robots traditionally fall back to autonomous and exploratory wayfinding operations. For humans, only recently decentralized collaborative wayfinding has been suggested [4]. Other research addressed this question mainly from the agents’ perspective. They deem the task as a Markov decision process or variations of it [5] and facilitate mission fulfilment by adapting and reacting to the uncertainty with strategies like multi-agent reinforcement learning, distributed learning algorithms [6,7].

Not much research can be found addressing the reliable shortest path problem from the perspective of a representation of the dynamics in the environment. Only from an economic perspective, Krek [8] has studied the impact of out-of-date data on decision making. In other research, the impact of the prediction of future states on decision making has been studied; results demonstrated a high sensitivity for the validity of the extrapolation [9,10]. The current paper, in contrast, suggests a novel approach: a map with fading memory by adding the temporal dimension to the acquired spatial knowledge. The paper presents an experiment to test the performance of shortest route planning considering the age of information in memory, putting less trust in older spatial knowledge and reports the results.

Fading memory devalues spatial knowledge with time, trusting recent explorations more than older ones. The hypothesis of this paper is that fading memory is beneficial for agents’ decision making in a dynamic environment. If this hypothesis is true, it has implications for all kinds of spatial analysis, but it also brings up questions of ethics: a service deliberately devaluing information that may in particular cases actually still be true is a service that accepts to produce in these cases suboptimal decisions [11] in favour of on average better decisions.

The remainder of this paper is organized as follows: Section 2 presents the related literature. In Section 3, a conceptual model for fading memory is described. Section 4 outlines the implementation of the simulation experiment, and in Section 5, results are presented and discussed. Finally, Section 6 concludes the work of this paper and proposes future work.

## 2. Related Work

Emergencies in indoor environment, such as fire, gas leaks and earthquakes, threaten occupants’ life and impact the evacuation routes by their own dynamics. As buildings are getting more complex, the risk attached to evacuation for occupants is increasing [12]. Time is critical for a successful evacuation; a delay of a few minutes may significantly increase the number of deaths [13]. Looking for effective evacuation strategies has been a long focus of research.

Techniques for indoor navigation have been reviewed by researchers from different perspectives, for example from locating the user, planning a path, representing the environment and interacting with the user perspective [14], from a context-aware perspective [15] or from a fire safety and human behaviour perspective [16].

Evacuees can be localized via QR code, radio frequency identification (RFID) [17], landmark detection [18], WiFi or other indoor localization techniques [19]. In this regard, indoor space is more complex than outdoor space. Furthermore, indoor space is more constrained by the built structure, is multi-level and is (mostly) private, with the corresponding access regulations. Effective representations of complex buildings are well researched. For example, Richter *et al.* [20] conceptualize indoor space into three hierarchical dimensions. Liu *et al.* [21] present a semi-automated method for identifying elements, such as hallways, elevators and stairways from 2D CAD files and constructing 3D building networks.

Route planning is the task to find an optimal route navigating a person from a current location to a destination while minimizing the travel time [22], travel distance or hazard [23,24]. Route planning in indoor space uses graphs [25] or grids [15] to represent the environment. Most of current navigation systems use Dijkstra [12,13] or A* algorithms [10]. The data for route planning can be stored and treated both in a local database [26] or a central database [27,28], which then requires a wireless connection to communicate the routes to evacuees.

While more complex buildings have been constructed to accommodate rapidly-growing population, development of emergency evacuation technology is relatively stagnant. Current evacuation support facilities are mainly limited to stationary exit signs and emergency maps, which are permanent without provision for *ad* *hoc* changes [3].

In recent years, the pervasive use of mobile devices, such as smart phones or tablet PCs, exhibits largely unexplored potential for more efficient evacuation. Since current emergency management and evacuation systems do not adapt information to each person, Aedo *et al.* [29] provide personalized alerts and evacuation routes to each evacuee. Smart phones are used as a support for escaping, instead of static signals on walls and doors. Smart phones will interact with the system, sending their current location and receiving multimodal messages personalized according to the emergency situation. In order to achieve a context-aware situation and provide personalized evacuation routes, Wang *et al.* [9] propose a framework for centralized evacuation, which combines a sensor system monitoring the whole environment with the route graph representing all of the possible routes. Evacuation routes are computed based on the original connectivity of indoor space and the data sensed in real time. Mobile devices are used for self-localization and visualizing the evacuation routes. This framework, although reflecting the best current knowledge, does not take into account that the evacuation routes may later be affected by the dynamics of the event. Even so, simulation suggests significantly better results than blind exploration. The framework has been improved by Wang *et al.* [10] through integrating also the temporal dimension and taking into account the influence that the dynamics of an event may have on the evacuation routes. A similar integrated real-time evacuation route planning method for high-rise building fires has been proposed by Han *et al.* [30]. Ahn and Han [22] have developed an indoor augmented reality system called RescueMe that runs on the users’ smart phones to guide people to evacuate from buildings in emergency situations. The system requires a communication between a smart phone and a cloud server via mobile social networking infrastructure. However, such centralized systems share a common shortcoming: a lack or a breakdown of the central infrastructure will lead to a failure of the whole system [31].

Only recently has decentralized evacuation management been studied. Decentralized evacuation management is scalable and robust to infrastructure failure [3], as well as ubiquitous [4]. Through agent-based simulations, Richter *et al.* [4] explore how cooperation and decentralization can improve the evacuation performance despite the prior knowledge of the agents in situations where central management or infrastructure has failed or was non-existing. Mobile devices are communicating in a peer-to-peer manner via short-range radio, and the shortest routes are calculated locally based on what is known about an environment by an individual at a time. They verify that decentralized evacuation management is nearly as successful as centralized management and independent from the state of the sensing and infrastructure in the building. However, without a central server, all local knowledge is time stamped, while the impact of the disaster on the environment may have outdated this knowledge.

So far, the task of decentralized evacuation route planning considering the uncertainty of information caused by the dynamics of events has received little attention from researchers. Recent research by Tan *et al.* [32] is distinguished from other works by considering not only the stationary environment during a normal situation, but also the event knowledge of predictable change in the spatial accessibility. However, the potentially changed spatial accessibility they refer to is caused by the activation of fire safety facilities, such as the fire rolling shutters during emergency scenarios. The validity of knowledge for other spatial connectivity due to time elapsed has not been addressed. To our knowledge the only literature that raises the question of uncertain information regarding evacuation in decentralized frameworks is by Merkel [3]. However, the uncertainty they refer to is caused by occasional disconnection of *ad hoc* networks caused by the movement through the building and the delay of communication incurred.

In an attempt to address the possibly out-of-date knowledge caused by the dynamics of expanding events in decentralized evacuation, this paper proposes a fading memory model, which represents not only the evacuees’ prior knowledge of the floor layout, but also the perceivable information about dynamic environment changes. System behaviour when introducing fading memory concepts will be tested via simulations.

## 3. System Model Formulation

This section introduces the definition of fading memory and describes how it can be applied in spatial decision making. The running examples are evacuation processes.

### 3.1. Fading Memory

Route planning for indoor evacuation can be accomplished by calculating the shortest route on the basis of a route graph *G* = (*V*, *E*), which consists of a set *V* = {*v*_1_, *v*_2_, ⋯, *v_K_*} of vertices and a set *E* = {*e*_1_, *e*_2_, ⋯, *e_N_*} of edges, where *K* is the total number of vertices and *N* is the total number of edges. The route graph embedded in ℝ^3^ draws a map of the walkable connectivity of all subspaces of the indoor environment. Different ways of deriving these route graphs for indoor environments have been proposed [33,34,35,36,37,38].

For centralized evacuation, the conditions of the environment can be sensed in real time by infrastructure; all of the evacuees share the same up-to-date knowledge maintained by a central server [39]. When sensing and communication infrastructure becomes unavailable, evacuees acquire knowledge of the environment through self-exploration, on the one hand, and communication with others, on the other hand [4]. In this case, the knowledge depends heavily on personal experience and may vary from person to person, such that each person computes their individual evacuation route based on their personal knowledge of the environment.

The critical information for route planning is whether a computed path is still passable or not. For example, an event may block a particular exit door or may have made a certain corridor unsafe. Independent from the type of event and related safety thresholds, e.g., for temperature, smoke or oxygen fraction, the passability of an edge in a route graph can be represented by a generic attribute called *state*. This attribute shall have two values: *blocked* and *unblocked*, where *blocked* means the space is unsafe and not passable.

Before an event, all of the edges of the route graph should be *unblocked*, and this state will be valid until the event start, which is when the states of some edges switch to being *blocked*, while others may follow later. During the evacuation, the state of the edges can be observed in various ways. In centralized systems, sensors will track passability, while decentralized systems encountering a blocked passage update the knowledge. Even an encounter with other evacuees can help: an evacuee can share with the encountering evacuee their individual knowledge of the environment, for example via short range radio communication on their smartphones (e.g., Bluetooth). A node in the route graph, representing a sub-space, can also be blocked by an event, but this case can be represented by setting all of the edges ending in this node as *blocked*.

#### 3.1.1. Definition

In this paper, the knowledge possessed by an evacuee for route planning is called the *route status map*, which consists of a set of triples called *memory segments*:

**Definition 1.**  A *memory segment* is an edge attached with an attribute describing its *state* and an attribute *t* describing the specific *time* when this memory segment has been acquired or has been last updated:

(1) *memseg* = (*e*, *state*, *t*)

where *e* denotes an edge of the route graph , state denotes the value of the state associated with that edge and *t* denotes the last time this memory segment was updated.

Let *E_m_* = {*memseg_i_*}; then, the route graph in memory is *G_m_* = (*V*, *E_m_*). Let also *t_cur_* denote the current time. Then, *t_cur_* − *t* indicates the risk that the state of an edge in memory might be out-of-date. With this, *fading memory* can be defined as:

**Definition 2.**  A *fading memory* $M_{fad}={\left(V,E_{m}\right)}_{t_{cur}}=\left(V,\left\{{\left(e,state,t_{cur}-t\right)}_{i}\right\}\right)$, where *i* ∈ {1, 2, ⋯, *N*}.

Thus, the fading memory *M_fad_* is an extended version of a route graph *G* representing the state of each edge and the age of the information about the state.

A memory collection can be represented by an attribute table for edges storing the attributes of each edge at time *t_cur_*. Table 1 shows an example of an attribute table for edges.

**Table 1:** Fading memory attribute table for edges, at an arbitrary time *t_cur_*.

Edge	State	Last Updated	Age
*e*_1_	*state*_1_	*t*_1_	*t_cur_* − *t*_1_
*e*_2_	*state*_2_	*t*_2_	*t_cur_* − *t*_2_
⋯	⋯	⋯	⋯
*e_n_*	*state_n_*	*t_n_*	*t_cur_* − *t_n_*

For simplicity, this paper assumes that before the event, people have a complete map of the environment, *i.e.*, a complete route graph. The fading memory attribute table will therefore cover all of the edges in the route graph.

During the event, the knowledge of an evacuee can be represented by a fading memory. From the components of the memory collection, fading memory *M_fad_* can be regarded as a map of the dynamic environment with three components. The edges compose the spatial components, describing the original topology of the spaces (before the event) for evacuation route planning. The states compose the dynamics of the environment, representing the update of the topology of the spaces during the event. The time composes the temporal components, describing how much the knowledge has been out of date. Thus, fading memory represents the spatial and temporal features of the updated knowledge of the dynamic environment.

In particular, if for all of the edges *t* = *t_cur_*, this fading memory is a *real-time memory*, which reflects the real-time conditions of the environment.

**Definition 3.**  A *real-time memory* consists of fading memory segments that are valid (sensed) at the current time, denoted by *M_real_*.

(2) *M_real_* = (*V*, {(*e*, *state*, 0)_*i*_})

where *i* ∈ {1, 2, ⋯, *N*}.

For decentralized evacuation, real-time memory is never available.

#### 3.1.2. Updating Fading Memory

The dynamic environment can be represented by a global route graph with all of the edge states sensed in real time (*i.e.*, a real-time memory). The knowledge of any evacuee can be represented by different local route graphs (or fading memory) possessed by each evacuee. The edge states of local route graphs coincide with the global route graph before the event. After the event, people share and update their knowledge in the form of memory segments. The fading memory can be updated in two ways:

- People acquire updated knowledge of the environment on a physical encounter with a changed environment. The memory segments acquired before the event or a longer while ago will be replaced by the memory segments that reflect the new observation of the encountered edges.
- People acquire updated knowledge of the environment on an encounter with fellow evacuees by exchanging their mutual time-stamped knowledge. Any memory segment that has a more recent counterpart in the encountered evacuee’s memory will be replaced by this more recent memory segment.

Evacuees update their local route graphs from their own trajectories when exploring the environment and from communication with fellow evacuees. These local route graphs are used by evacuees to plan evacuation paths.

### 3.2. Routing with Fading Memory

Fading memory is represented by an attributed graph highlighting not only the connectivity of the space, but also representing the elapsing time after acquiring that knowledge. Thus, routing can be a standard *k*-shortest path problem, labelling edges known to be blocked by infinite costs. The *k* alternative paths are then assessed by trusting more the recently acquired knowledge and less the knowledge that has been explored a longer time ago. Spatial factors, such as the location of the blocked edges, can also be taken into consideration, for example, by preferring paths that do not not come near blocked edges.

**Figure 1:**
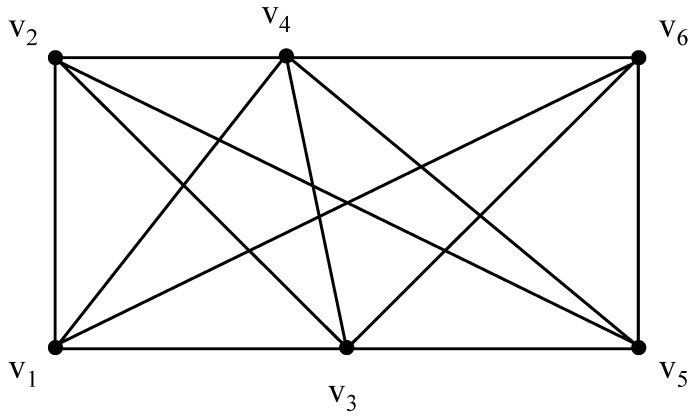
A sample route graph.

Threshold-based assessment methods have been used in a number of studies (e.g., to detect human faces from colour images [40]). To demonstrate how a time-aware routing map can be achieved with fading memory, an evolving threshold-based method is deployed here exemplarily. This method can be replaced by any other reasonable method. For demonstration, a sample route graph with six nodes is illustrated in Figure 1. The original connectivity can be represented by Table 2, where the cell {*v_i_*, *v_j_*} = 1 if there is an edge linking *v_i_* and *v_j_*, else {*v_i_*, *v_j_*} = 0; *i*, *j* ∈ {1, 2, ⋯, 6}.

**Table 2:** The connectivity of the sample route graph.

*G*	*v*_1_	*v*_2_	*v*_3_	*v*_4_	*v*_5_	*v*_6_
*v*_1_	0	1	1	1	0	1
*v*_2_	1	0	1	1	1	0
*v*_3_	1	1	0	1	1	1
*v*_4_	1	1	1	0	1	1
*v*_5_	0	1	1	1	0	1
*v*_6_	1	0	1	1	1	0

The states of the edges in the route graph can be represented by Table 3, where the cell {*v_i_*, *v_j_*} contains the state for edge {*v_i_*, *v_j_*}. Before the event, all edges have status *u*, denoting an *unblocked* state.

Suppose the event starts at *t_event_*. Since then, an evacuee *P*_1_ acquires some additional knowledge, be it through self-exploration or peer-to-peer knowledge exchange, and updates the corresponding four segments in his or her memory as in Table 4 (assume *t_event_* < (*t*_1_, *t*_2_, *t*_3_, *t*_4_) < *t_cur_*). The corresponding updated route graph is shown in Table 5 (*b* denotes a *blocked* state).

**Table 3:** The original states of the sample route graph.

	*v*_1_	*v*_2_	*v*_3_	*v*_4_	*v*_5_	*v*_6_
*v*_1_		u	u	u		u
*v*_2_	u		u	u	u	
*v*_3_	u	u		u	u	u
*v*_4_	u	u	u		u	u
*v*_5_		u	u	u		u
*v*_6_	u		u	u	u	

**Table 4:** The attribute table for the fading memory at a time *t_cur_*, with four updated memory segments.

Edge	State	Last Updated	Age
{*v*_1_, *v*_2_} & {*v*_2_, *v*_1_}	*blocked*	*t*_1_	*t_cur_* − *t*_1_
{*v*_1_, *v*_3_} & {*v*_3_, *v*_1_}	*unblocked*	*t*_2_	*t_cur_* − *t*_2_
{*v*_3_, *v*_4_} & {*v*_4_, *v*_3_}	*unblocked*	*t*_3_	*t_cur_* − *t*_3_
{*v*_1_, *v*_4_} & {*v*_4_, *v*_1_}	*unblocked*	*t*_4_	*t_cur_* − *t*_4_
{*v*_1_, *v*_6_} & {*v*_6_, *v*_1_}	*unblocked*	*t_event_*	*t_cur_* − *t_event_*
{*v*_2_, *v*_3_} & {*v*_3_, *v*_2_}	*unblocked*	*t_event_*	*t_cur_* − *t_event_*
{*v*_2_, *v*_4_} & {*v*_4_, *v*_2_}	*unblocked*	*t_event_*	*t_cur_* − *t_event_*
{*v*_2_, *v*_5_} & {*v*_5_, *v*_2_}	*unblocked*	*t_event_*	*t_cur_* − *t_event_*
{*v*_3_, *v*_5_} & {*v*_5_, *v*_3_}	*unblocked*	*t_event_*	*t_cur_* − *t_event_*
{*v*_3_, *v*_6_} & {*v*_6_, *v*_3_}	*unblocked*	*t_event_*	*t_cur_* − *t_event_*
{*v*_4_, *v*_5_} & {*v*_5_, *v*_4_}	*unblocked*	*t_event_*	*t_cur_* − *t_event_*
{*v*_4_, *v*_6_} & {*v*_6_, *v*_4_}	*unblocked*	*t_event_*	*t_cur_* − *t_event_*
{*v*_5_, *v*_6_} & {*v*_6_, *v*_5_}	*unblocked*	*t_event_*	*t_cur_* − *t_event_*

**Table 5:** The states of the route graph after state propagation from the fading memory.

	*v*_1_	*v*_2_	*v*_3_	*v*_4_	*v*_5_	*v*_6_
*v*_1_		b	u	u		u
*v*_2_	b		u	u	u	
*v*_3_	u	u		u	u	u
*v*_4_	u	u	u		u	u
*v*_5_		u	u	u		u
*v*_6_	u		u	u	u	

This updated fading memory can be used for route planning. The following steps describe the process applying an evolving threshold-based route assessment approach:

1 Step 1: Initialize the route graph. Propagate the current states in the fading memory to the route graph (Table 5).
  The next step (2a,b) is optional and introduces a mechanism to avoid edges known to be unblocked if they are near blocked edges and the knowledge of being unblocked is quite old, *i.e.*, the risk that they are no longer unblocked is high.
2 Step 2a: Considering the spatial features, label the states of some edges as *c* (for further checking): these are the edges that are currently marked as *u* for *unblocked*, but are directly connected with one or more *blocked* edges.
  Table 6 shows the updated state table for the running example: the edge that has a state of *blocked* in fading memory is {*v*_1_, *v*_2_}; thus, all of the edges that end either in *v*_1_ or *v*_2_ and are currently *unblocked* are labelled *c*.
  Step 2b: Considering the temporal features, revise the states of some edges with a threshold. For all edges that are labelled *c*, check the age of the knowledge of their states: if the age exceeds a threshold (initialled with a predefined threshold denoted by *Th*), then their states will be revised to *blocked*, otherwise their state will be reverted to *unblocked*.
  In the running example, suppose *t_event_* = 0, *t_cur_* = 0, *Th* = 20, and *t*_1_, *t*_2_, *t*_3_ and *t*_4_ have some value between *t_event_* and *t_cur_*. Then, the attribute table will look like Table 7. Note how the states of edges {*v*_1_, *v*_4_}, {*v*_4_, *v*_1_}, {*v*_1_, *v*_6_}, {*v*_6_, *v*_1_}, {*v*_2_, *v*_3_}, {*v*_3_, *v*_2_}, {*v*_2_, *v*_4_}, {*v*_4_, *v*_2_}, {*v*_2_, *v*_5_} and {*v*_5_, *v*_2_} have been revised to *blocked* and {*v*_1_, *v*_3_}, {*v*_3_, *v*_1_} have been reverted to *unblocked* (Table 8).
3 Step 3: Update the route graph and compute the evacuation path. Set the weight for the edges labelled *b* as infinity. Set the weight for the edges labelled *u* by any cost function, for example one (for routes of the fewest legs) or their actual length (for routes of the shortest distance). Calculate the shortest path based on the weights of the edges in the route graph.
4 Step 4: Adapt the threshold in case no evacuation path is available due to the potential overestimation of the severity of the event. If the shortest path calculated from Step 3 contains blocked edges or edges that have been checked as blocked, adapt the threshold by setting the threshold with a larger value, and go to Step 2b. If the adaptive threshold exceeds a limitation and still no passable path can be found, the evacuee is considered as failing in evacuation.

**Table 6:** The states of the route graph after marking the states that need further checking.

	*v*_1_	*v*_2_	*v*_3_	*v*_4_	*v*_5_	*v*_6_
*v*_1_		b	c	c		c
*v*_2_	b		c	c	c	
*v*_3_	c	c		u	u	u
*v*_4_	c	c	u		u	u
*v*_5_		c	u	u		u
*v*_6_	c		u	u	u	

**Table 7:** The attribute table with sample value when *t_cur_* = 40.

Edge	State	Last Updated	Age
{*v*_1_, *v*_2_} & {*v*_2_, *v*_1_}	*blocked*	10	30
{*v*_1_, *v*_3_} & {*v*_3_, *v*_1_}	*unblocked*	25	15
{*v*_3_, *v*_4_} & {*v*_4_, *v*_3_}	*unblocked*	30	10
{*v*_1_, *v*_4_} & {*v*_4_, *v*_1_}	*unblocked*	15	25
{*v*_1_, *v*_6_} & {*v*_6_, *v*_1_}	*unblocked*	0	40
{*v*_2_, *v*_3_} & {*v*_3_, *v*_2_}	*unblocked*	0	40
{*v*_2_, *v*_4_} & {*v*_4_, *v*_2_}	*unblocked*	0	40
{*v*_2_, *v*_5_} & {*v*_5_, *v*_2_}	*unblocked*	0	40
{*v*_3_, *v*_5_} & {*v*_5_, *v*_3_}	*unblocked*	0	40
{*v*_3_, *v*_6_} & {*v*_6_, *v*_3_}	*unblocked*	0	40
{*v*_4_, *v*_5_} & {*v*_5_, *v*_4_}	*unblocked*	0	40
{*v*_4_, *v*_6_} & {*v*_6_, *v*_4_}	*unblocked*	0	40
{*v*_5_, *v*_6_} & {*v*_6_, *v*_5_}	*unblocked*	0	40

**Table 8:** The states of the route graph after further checking.

	*v*_1_	*v*_2_	*v*_3_	*v*_4_	*v*_5_	*v*_6_
*v*_1_		b	u	b		b
*v*_2_	b		b	b	b	
*v*_3_	u	b		u	u	u
*v*_4_	b	b	u		u	u
*v*_5_		b	u	u		u
*v*_6_	b		u	u	u	

**Figure 2:**
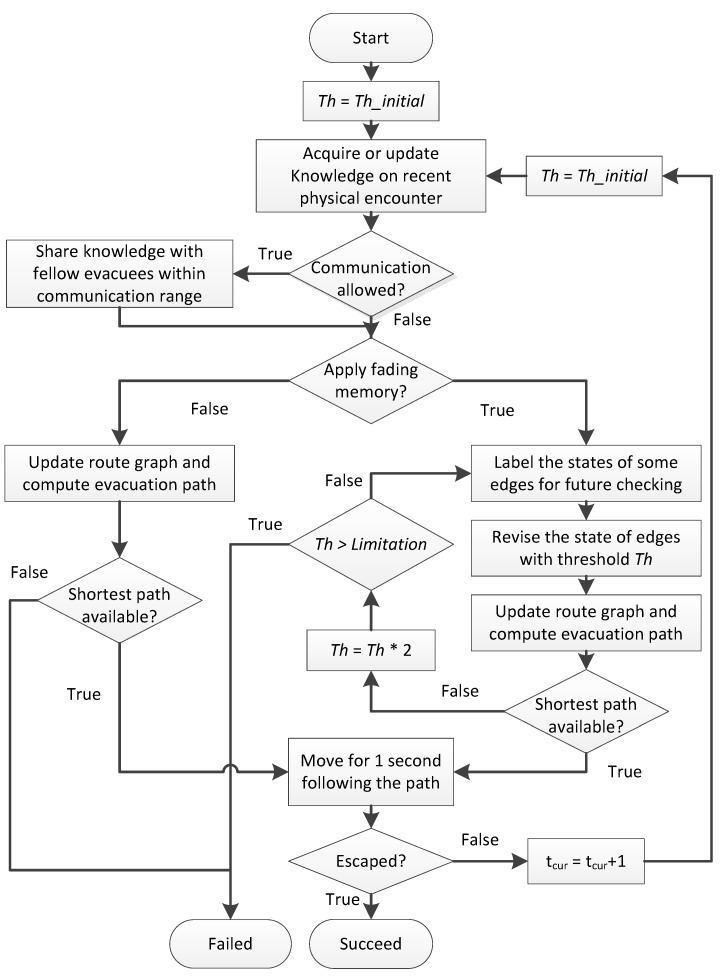
The process of the evacuation with fading memory.

The evolving routing method with fading memory considers the spatial and temporal factors of the knowledge acquired, considering the age of the spatial knowledge. The out-of-date knowledge can only be tolerable when no evacuation path is available due to conservatism. Even with the threshold being adapted in each step, the initial threshold can still affect the route planning by biasing to choose a path that is a detour, but is deemed to be safer if more than one path is available. The simulation process can be illustrated with the flow chart in Figure 2.

### 3.3. A Generic Method

The threshold-based approach above is relatively crude in its consideration of the age of acquired information. A more generic method to apply fading memory in evacuation management is to use a two-step method for routing.

In the first step, the states and the age of the acquired knowledge in fading memory will not be considered. Instead, a standard *k*-shortest path algorithm is applied, on the static route graph, calculating a list of shortest paths. Such *k*-shortest path algorithms have been studied by many scholars [41,42,43,44,45,46]. A classical loopless *k*-shortest path algorithm is described by Yen [41] and used here.

Only in the second step the fading memory exhibits its influence. The second step assesses the alternative *k* shortest paths for their likelihood to be unblocked, trusting more the recently-acquired knowledge. The assessment of a path can be represented by any suited evaluation function *fade*(), such that *fade*(*Path^i^*) ≥ 0, where *Path^i^* is the *i*-th shortest path (*i* ≤ *k*). *fade*(*Path^i^*) denotes the trust that path *Path^i^* is unblocked according to the fading memory and, thus, can be recursively defined:

(3) $$fade\left({Path}^{i}\right)=\text{min}\left\{fade\left(e_{j}^{i}\right)\right\}$$

The selection of a route (e.g., by an evacuee) can then be based on balancing the length of the route and the trustworthiness of the route. For example, in life-threatening scenarios, one will accept any detour and choose the route of lowest risk. This consideration also suggests that the evaluation function itself has to be chosen carefully for particular applications. It may depend on the type of event (how fast it spreads) or, more generally, on the dynamicity of the environment. The evolving threshold-based approach above is one example for an evaluation function:

(4) $$fade\left(e_{j}^{i}\right)=Th-{\left(t_{cur}-t\right)}_{j}$$

where *Th* is the threshold, and a value of *fade* < 0 is considered blocked. The threshold *Th* is first set with an initial value and then is adaptive to avoid potential overestimation of the severity of the event.

## 4. Experiments

The concept of fading memory has been implemented in an agent-based simulation of an emergency event and consecutive evacuation. The experiment has been designed to test and compare the success rates of evacuations with and without fading memory.

The simulation is designed to test the system behaviour when introducing the concept of fading memory. Thus it suffices to assume that evacuees follow the aforementioned evacuation strategies during the evacuation, *i.e.*, follow route instructions of their mobile devices that maintain the fading memory and compute optimal routes. Thus, the simulation does not aim to predict human behaviour, which also has been considered almost impossible elsewhere [47]. It does also not aim to simulate a particular event-spreading process, such as a fire in a building [48,49], but could be fed with particular spreading models.

Events at different levels of speed have been simulated. For each level of speed, 60 different events are simulated, recorded and recalled when comparing evacuation scenarios, such that each comparison refers to the same event.

### 4.1. Event Simulation

For the experiment, a spatially-extended and temporally-varying event has been assumed, for example a fire breaking out in a building, starting from a single location and continuing to expand. Events of other characteristics, such as earthquakes that impact at multiple locations at the same time or bomb explosions that do not expand after going off, will not be covered by this particular experiment, but could be tested in the same way in differently-designed simulations. At the beginning of an event, some edges in the route graph will be affected and become blocked. Then, the event expands, affecting more edges and causing them to be blocked. Because of the continuity of the event spreading, the effected subspaces in the indoor environment are adjacent and connected (*i.e.*, not separated by walls).

In order to simulate the event, this paper adopts the concept of sensor graphs described by Wang *et al.* [9]. The real-time situation of the environment is assumed to be monitored by a virtual sensor network that covers the whole area of the indoor environment. The sensor network is generated by adding an edge between two sensor nodes if their detecting areas are adjacent and connected. Two sensor nodes are neighbouring each other if they are connected with an edge. One sensor is set to be active from the beginning. Then, because of the continuity of the event spreading, the next active sensor should be a neighboured sensor of the already active one. Thus, an extended process can be simulated through simulating the state transfer of the sensors. The advantage of modeling the event independently from the route graph is obvious: the sensors can be distributed equidistantly and can cover also spaces that are not directly covered by the route graph.

In this paper, a sensor shall have two possible states, *normal* (denoted by *N*) and *active* (denoted by *A*). A sensor stays in the *normal* state when its detecting area is safe and passable and may shift to *active* when its detecting area becomes unsound because of the event. Before the event, all sensors should be in the *normal* state, then one sensor that covers the area where the event starts shifts to *active*. As the event expands, for any neighboured sensor of an *active* sensor, a state transfer from *normal* to *active* may happen not definitely, but with a certain probability (denoted by *p*), which depends on the speed of the event expansion. A larger value of the probability *p* means a faster expanding event. In the implemented simulation, every 10 s, the event is updated by selecting the neighboured sensors of a randomly-picked active sensor and will be set active with a certain probability. This way, 60 events have been recorded, such that different memory strategies can be applied to the same events.

### 4.2. Evacuation Strategies

For simplicity, this experiment assumes that all people are acquainted with the environment before the event: they have complete knowledge of the (static) route graph. In addition, this experiment assumes that during an event, people acquire knowledge and update their memory applying the two mechanisms mentioned above:

- People acquire updated knowledge of the environment upon a physical encounter with a changed environment.
- People acquire updated knowledge of the environment upon an encounter with fellow evacuees by exchanging their mutual time-stamped knowledge.

Each evacuee computes their own evacuation routes based on their own memory of the environment. The routes will be adjusted each time when their memory of the environment is updated. Routes will be computed applying the steps described in Section 3.2. Since the communication channel may be blocked, considering whether peer-to-peer communication is allowed and whether dynamic routing with fading memory is applied, strategies that can be applied by evacuees are classified into four categories:

- **FS**: apply fading memory for dynamic route planning; communication is allowed so that evacuees share knowledge.
- **CS** : not applying fading memory, but communication is allowed.
- **FN**: the same as **FS**, except that communication is not allowed, so that knowledge cannot be shared.
- **CN**: The same as **CS**, except that communication is not allowed, so that knowledge cannot be shared.

Although the movement velocity of elderly people, young children or those with some form of impairment varies, in this experiment, we assume a constant speed of 1.5 m/s. When the fire alarm is set off, all occupants start to evacuate. If no evacuation path is available for an evacuee, this evacuee is declared as failing. Depending on the circumstances, such evacuees may survive, e.g., if the fire is extinguished before it reaches them; however, in the context of this experiment, only the successfully evacuating agents are counted.

Both the models of event spreading and the evacuation of agents with fading memory have been implemented and tested in Repast Simphony [50]. The experiment adopts a five-floor office building as a sample evacuation environment and generates the route graph of this building with YAMAMOTO [37]. One hundred evacuees are placed at predefined locations when the events start and then walk following the path generated from the route graph during evacuation. For comparability, these evacuees always start at the same locations; only the seed location and (random) spread of the event varies from simulation to simulation.

## 5. Results and Discussions

### 5.1. Experiment 1: Communication Allowed

Experiments test the evacuation performance of the 100 evacuees with **FS** and **CS** under 60 emergency events with the initial threshold selected from a list of values: 1, 5, 10, 20, 40, 80, 160, 320. When all of the evacuees reach an exit or have no path left to evacuate, the evacuation process is considered as completed.

**Figure 3:**
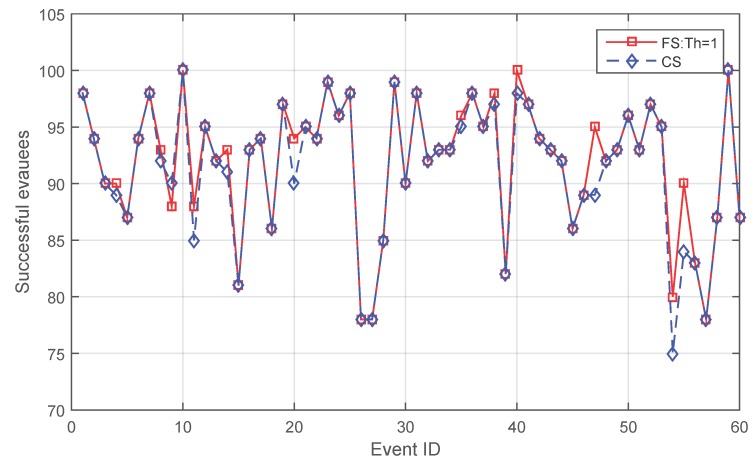
Comparison between **FS** and **CS** when the initial threshold is one.

Figure 3 shows that when the threshold is set as one, in most cases (49 cases), the effect of applying fading memory leads to neither an increase nor a decrease in the quantity of successful evacuees. However, for the rest of the cases, fading memory outperforms **CS** in 10 cases, saving 3.2 people more on average per case. **CS** is better only in one case (Case 9), saving two people more. When examining the details of Case 9, both of the two people fail because of a sudden change of the environment. As described in Section 4.1, the event expands in the simulation every 10 s, which is not a smoothly-changing environment. The two people have first selected a longer path due to a rigid initial threshold of one, then suddenly failed because the event expanded and blocked the edges in which the two people were standing. In contrast, for the corresponding **CS** strategy, the two people have first chosen a shorter path and just avoided standing in a blocked edge when that event suddenly expanded.

**Figure 4:**
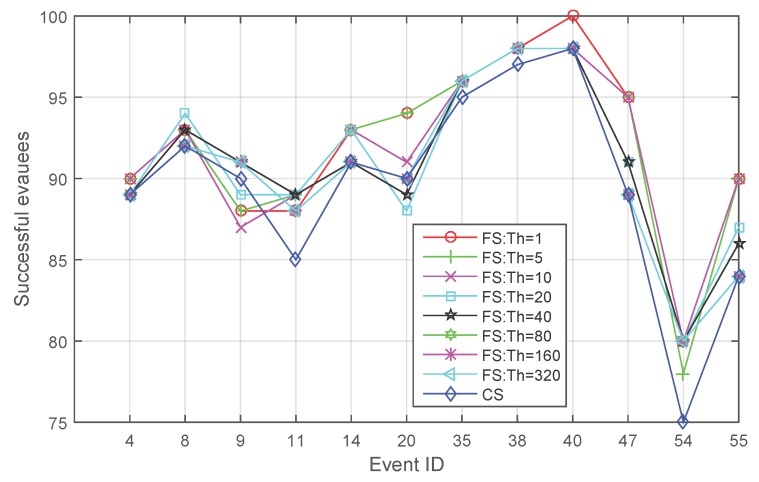
Comparison between **FS** and **CS** for the event cases where the quantity of people saved by different evacuation strategies exhibits differences.

Figure 4 compares those event cases where the quantity of people saved by different evacuation strategies exhibits differences when applying varying initial thresholds for **FS**. **CS** maintains the lowest success in 10 events. Only in two cases (Cases 9 and 20), **CS** is no longer the lowest one and is not the highest one either in the number of successful evacuees. Comparing Figure 3 and Figure 4, what can be observed is that even in one case (Case 9), **CS** saves two people more, but the advantage of **CS** is not maintained when the initial threshold for **FS** changes. When the initial threshold gets larger (for example, 40), **FS** again saves more people.

**Figure 5:**
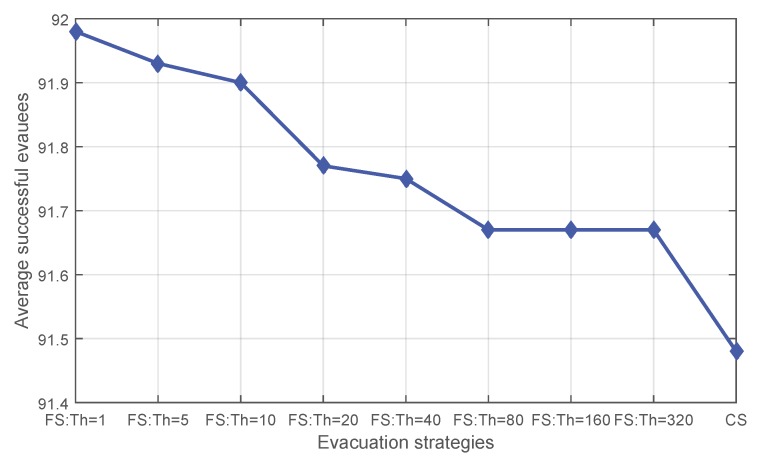
The average number of successful evacuees for **CS** and **FS** with varying initial thresholds.

Figure 5 shows the average success drawn from the 60 events when applying different evacuation strategies. Applying fading memory always saves a larger average number of evacuees. When the initial threshold gets larger, the effect of fading memory decreases.

### 5.2. Experiment 2: Communication Not Allowed

**Figure 6:**
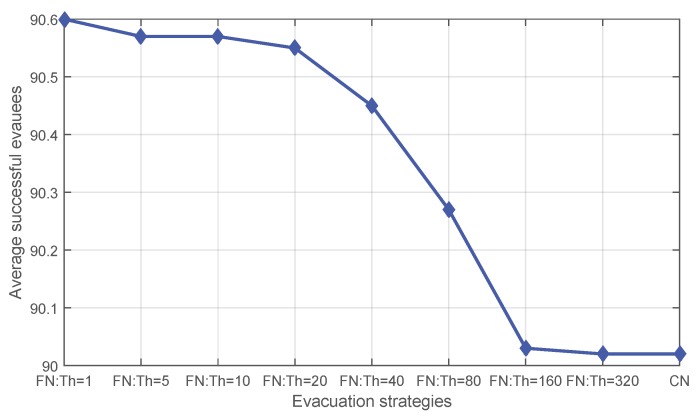
The average number of successful evacuees for **CN** and **FN** with varying initial thresholds.

**Figure 7:**
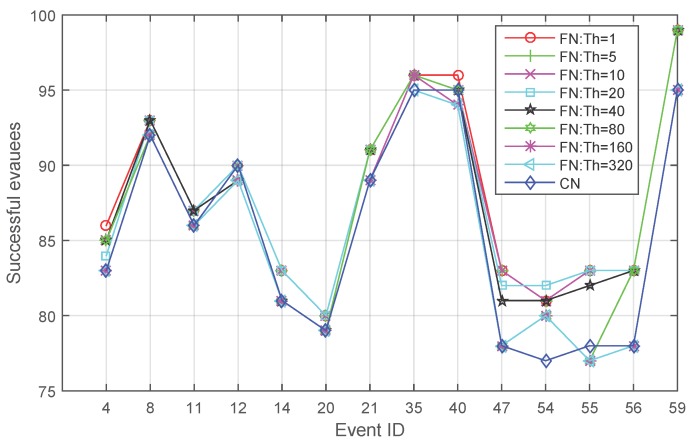
Comparison between **FN** and **CN** for the event cases where the quantity of people saved by different evacuation strategies exhibits differences.

Peer-to-peer communication can be achieved by using mobile devices [4]. However, even short-range communication channels may not exist (e.g., on smartphones with no Bluetooth). In order to investigate whether applying fading memory is still beneficial independent of any communication channel, experiments have also tested the performance of **FN** with varying initial thresholds applied when peer-to-peer communication is not allowed. Results show a similar pattern in the average number of successful evacuees (Figure 6). On average, fading memory exhibits an advantage. This advantage diminishes as the initial threshold increases until approaching a limit at which the performance of the threshold-based method equals that without applying fading memory. This is reasonable, because when the initial threshold gets larger, more out-of-date knowledge will be trusted, and in particular, when the initial threshold exceeds the evacuation time of all evacuees, fading memory degrades not applying fading memory. Figure 7 compares the event cases where the quantity of people saved by different evacuation strategies exhibits differences. Fading memory shows better performance than **CN** in 11 events, while only in three cases, a varying initial threshold fails to guarantee saving more people than **CN**.

### 5.3. Experiment 3: Sensitivity Test for Event Speed

The probability *p* of sensor state transfer adopted in the event simulation reflects the speed of the expansion of an event. In Experiments 1 and 2, a transfer probability of 100% has been adopted, but this probability *p* is an arbitrary choice. In order to test whether the simulation result is sensitive to the selection of this parameter, the previous experiment has been repeated, but the sensors update their states with probabilities selected from a series of values: 20%, 40%, 60%, 100% and 0%.

**Figure 8:**
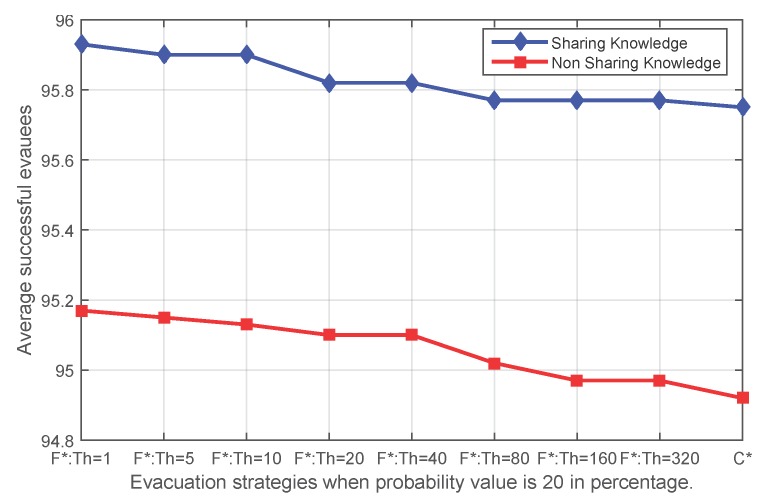
The average number of successful evacuees when the probability value of the sensor state transfer adopted in the event simulation is 20% (F* corresponds to **FS** and **FN**; C* corresponds to **CS** and **CN**).

**Figure 9:**
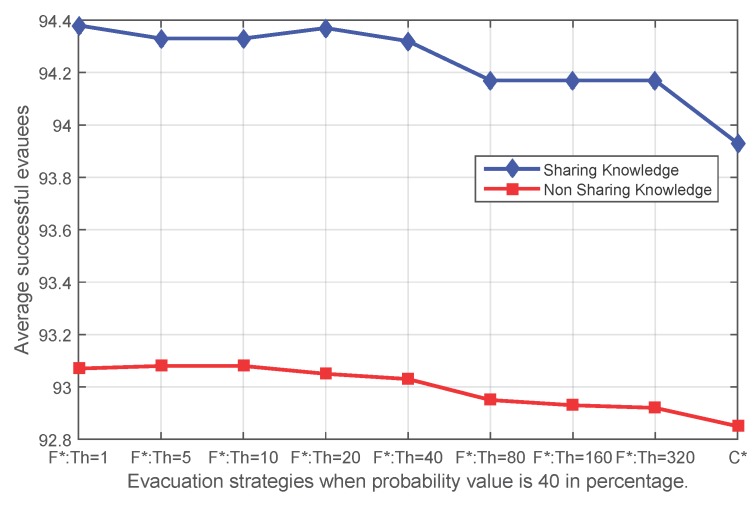
The average number of successful evacuees when the probability value of the sensor state transfer adopted in the event simulation is 40% (F* corresponds to **FS** and **FN**; C* corresponds to **CS** and **CN**).

**Figure 10:**
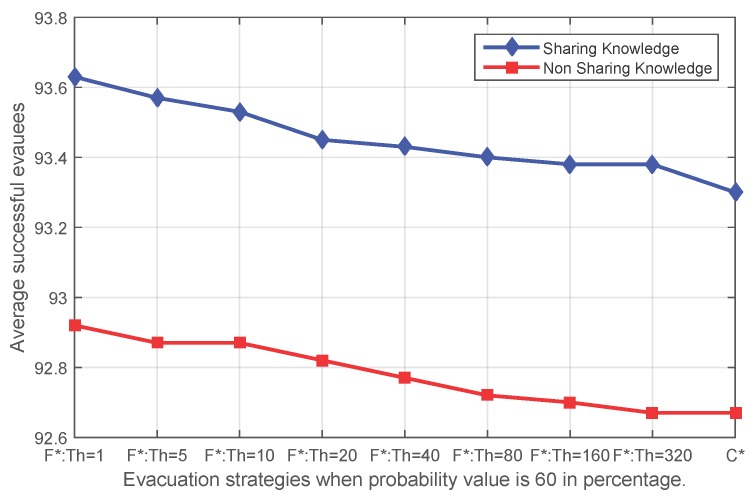
The average number of successful evacuees when the probability value of the sensor state transfer adopted in the event simulation is 60% (F* corresponds to **FS** and **FN**; C* corresponds to **CS** and **CN**).

**Figure 11:**
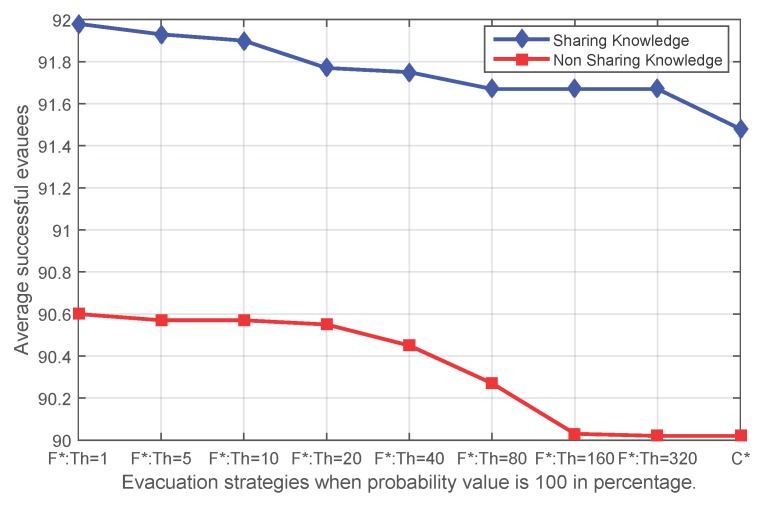
The average number of successful evacuees when the probability value of the sensor state transfer adopted in the event simulation is 100% (F* corresponds to **FS** and **FN**; C* corresponds to **CS** and **CN**).

Figure 8, Figure 9, Figure 10 and Figure 11 show that independent of the expansion speed of an event, the quantity of average success shows a similar pattern. Applying fading memory saves on average more people than without applying fading memory. With growing thresholds, this advantage diminishes until the success rates reach the same outcomes as without applying fading memory. Since a high probability *p* means a faster expanding event and, thus, less opportunity for evacuating, it is reasonable that when the probability *p* in the event simulation is lower (for example 20%), more people have been safely evacuated than with higher probabilities, whether applying fading memory or not.

**Figure 12:**
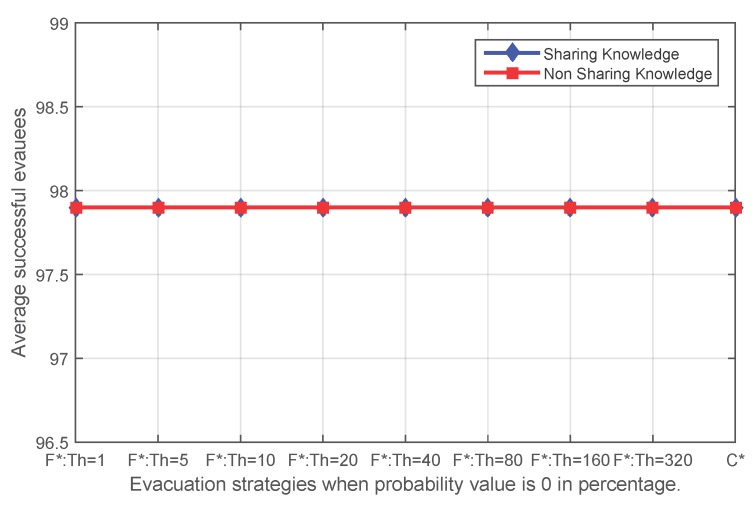
The average number of successful evacuees when the probability value of the sensor state transfer adopted in the event simulation is 0% (F* corresponds to **FS** and **FN**; C* corresponds to **CS** and **CN**).

In particular, when the probability value decreases to zero, the event becomes a static event, an event that affects some edges in the route graph and does not expand. When the dynamic event degrades to a static event, the topology and states of the route graph will remain constant. Thus, all of the knowledge acquired by evacuees will always be valid, represents the real-time circumstance of the environment and should be completely trusted. For this particular event, applying fading memory is expected to make no improvements in the number of successful evacuees, but whether it deteriorates the result needs to be tested. Experiment results for a static event show that exactly the same number of evacuees will be saved no matter whether fading memory has been applied and no matter what initial threshold has been applied for the route planning with fading memory (Figure 12).

The sensitivity test for different speeds of events indicates that the advantages of fading memory are maintained across events of all speeds and, in particular, is reliable even for static events. The dynamic routing method with fading memory is capable of guaranteeing a higher average of successful evacuees.

### 5.4. Experiment 4: Comparison with Real-Time Knowledge

Evacuees acquire and update knowledge through exploration and communication with fellow evacuees. The knowledge of an evacuee has been represented by a fading memory so far, which is a local route graph with time-stamped states. Fading memory draws a map of the dynamic environment and can be applied to achieve an evolving dynamic route planning with spatial and temporal factors taken into consideration. The fading memory for different evacuees may differ due to their varying trajectories. As mentioned in Section 3.1.2, the real-time conditions of the environment can be represented by a real-time memory, which is a global route graph with all states sensed in real time. In the decentralized evacuation process, the global route graph is always unique and is not accessible for evacuees. However, in centralized evacuation, the real-time knowledge can be sensed and computed in a central infrastructure [9]. Based on the real-time information of the environment, personalized evacuation paths can be computed and sent to each evacuee via mobile devices.

**Figure 13:**
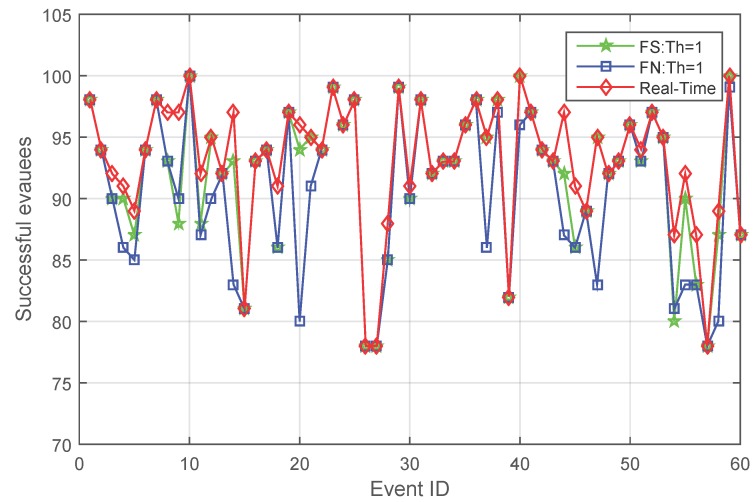
Comparison of the number of successful evacuees between centralized evacuation and decentralized evacuation with fading memory both when knowledge is shared and not shared.

The evacuation results of centralized evacuation planning with real-time knowledge are a benchmark for decentralized evacuation with fading memory. In order to investigate the gap between fading memory and a centralized evacuation, this last experiment compares the centralized evacuation implemented by Wang *et al.* [9] and evacuations with fading memory. Figure 13 compares the performance of centralized evacuation and decentralized evacuation with fading memory, both when knowledge is shared and not shared.

As can be observed from Figure 13, although fading memory is capable of improving evacuation success in decentralized evacuation, it is not likely to be better than the evacuation performance of centralized evacuation. From the experiment results, centralized evacuation always saves at least the same, but sometimes more people than fading memory.

However, in a significant number of cases, fading memory results in saving the same number of successful evacuees. When examining the details of the results, this is because a number of evacuees failed to find an evacuation path even at the beginning of an evacuation due to a lack of alternatives; then, different evacuation strategies make no difference.

## 6. Conclusions

Spatial knowledge of dynamic environments fades in its value for decision making because of the growing risk that it has become invalid over time. This paper proposes a fading memory model to represent the knowledge of a dynamic environment and applies it to route planning. Results show that this fading memory model can improve the evacuation success rates for expanding events or in general improve the decisions made in a dynamic environment.

With this evidence at hand, this paper is the first approach to fading memory and a beginning to the full exploration of the properties and promises of fading memory on decision making in dynamic environments. The simulation in this paper leaves aside any human behavioural factors, such as walking speed, visual ability or acting under stress. It also does not consider congestions caused by evacuee crowds, which should be taken into account in evacuation guidance in practice. Similarly, the prior knowledge of evacuees has so far been assumed to be complete; however, this is most likely not the case in real-world situations in complex indoor environments. Thus, studying the evacuation from an environment with limited (or no) prior knowledge and fading memory is also part of future work. The experiments in this paper are completed based on a sample building with limited alternatives. More generic graphs that represent different dynamic environments can also be tested. The density of evacuees in the experiment is limited, and a higher density of evacuees means that more opportunity for communication and that more space can be covered by the trajectories of evacuees in a short time; thus, this may lead to higher efficiency of evacuation. The discontinuity of the event expanding in the simulation is noise for the simulation results, which can also be improved in future work. Finally, the assessment model can be refined. For example, a formulation by probabilities of blocked edges can replace a threshold model more elegantly, and probabilities can be produced according to the type (or aggressiveness) of the event, say fire spread compared to gas spread.
